# Laparoscopic Management of Esophageal Epiphrenic Diverticulum

**DOI:** 10.7759/cureus.66663

**Published:** 2024-08-12

**Authors:** Shantanu S Navgale, Satish B Dharap, Sampada Wankhede, Prabhakar Guvvala

**Affiliations:** 1 General Surgery, Topiwala National Medical College and Bai Yamunabai Laxman Nair Charitable Hospital, Mumbai, Mumbai, IND

**Keywords:** antireflux surgery, heller’s myotomy with dor fundoplication, diverticulectomy, laparoscopic heller's cardiomyotomy, epiphrenic esophageal diverticulum

## Abstract

A 45-year-old man who presented with progressive dysphagia of five months duration was diagnosed as a case of oesophageal epiphrenic diverticulum after endoscopic and imaging investigations. He underwent laparoscopic cardiomyotomy with Dor's fundoplication. Myotomy was done from the base of the diverticulum up to 2 cm distal to the gastroesophageal junction. Intraoperative endoscopy was done to check the adequacy of myotomy. Diverticulectomy was not done. Yet the patient had complete relief of symptoms and is well and asymptomatic after two years. Cardiomyotomy with anti-reflux procedures is effective in treating the epiphrenic diverticulum without the need for resection of the diverticulum, which also provides a better prognosis and less morbidity to the patient.

## Introduction

The esophageal diverticulum is a rare structural abnormality of the esophagus. The mechanisms thought are “push” from within (pulsion occurs due to raised intraluminal pressure from a distal high-pressure zone) or “pull” from outside (traction). Cervical and epiphrenic diverticula are considered pulsion diverticulums with mucosal and submucosal herniation due to the high-pressure zone in the immediate distal esophagus. Midthoracic diverticula are often secondary to traction exerted by a mediastinal inflammatory process and involve all the layers of the esophageal wall [1]. Surgical management is the treatment of choice in patients with symptomatic diverticulum. The literature review has reported a high incidence of postoperative morbidity [1-3].

Here, we describe a case of epiphrenic esophageal diverticulum with progressive dysphagia, which was successfully treated with laparoscopic Heller’s cardiomyotomy and Dor’s fundoplication, without the need for diverticulectomy.

## Case presentation

A 45-year-old male presented with progressive dysphagia, retrosternal, and epigastric burning sensation of five months duration. Initially, dysphagia was for liquids and progressed to affect solid intake as well. The symptoms had worsened in the previous three months with a weight loss of 10 kgs. He had been a chronic smoker for 20 years without any other comorbidity.

**Figure 1:**
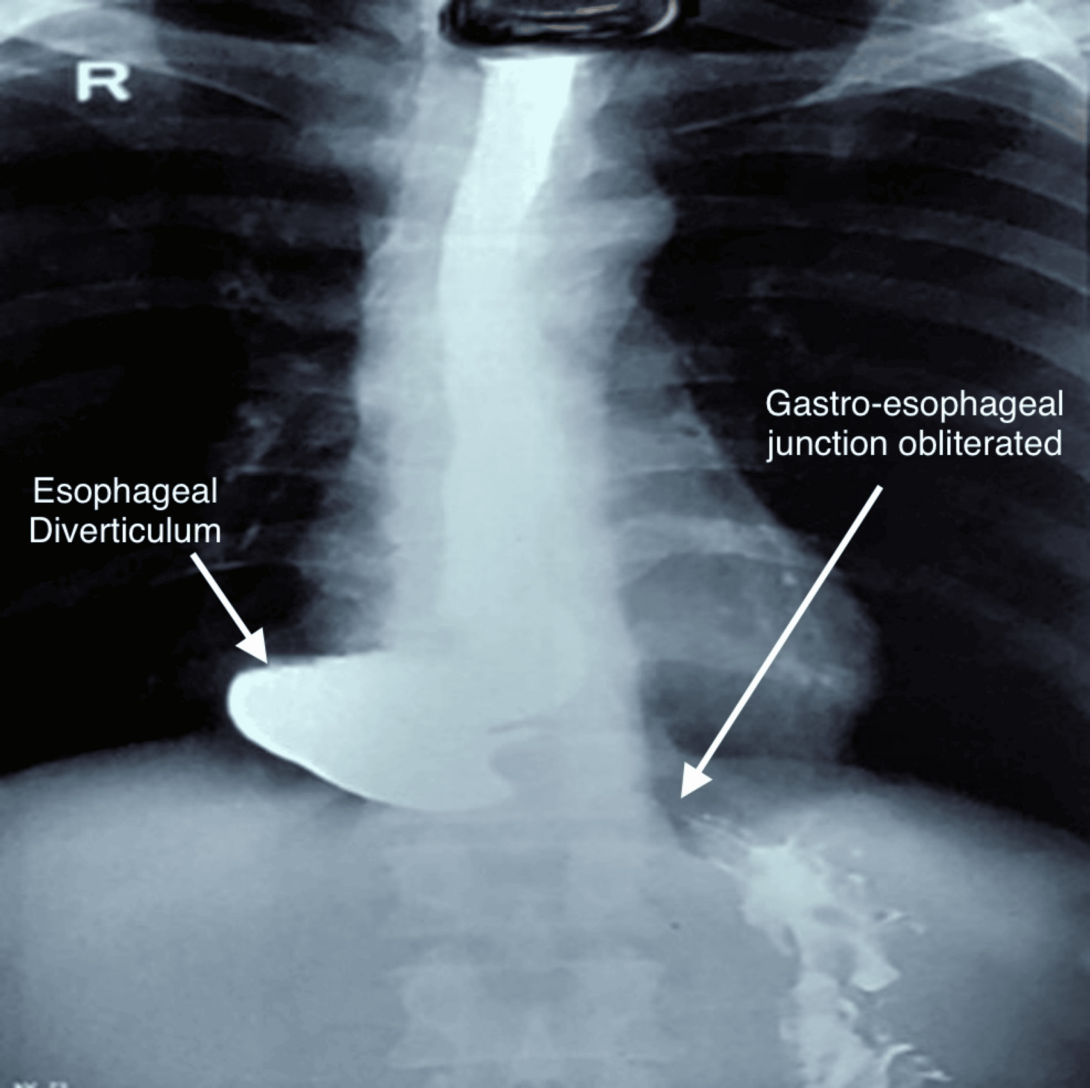
Preoperative barium swallow study showing dilatation of the esophagus and the presence of epiphrenic diverticula. Oral contrast fails to pass through the gastroesophageal junction.

An upper gastrointestinal barium swallow study revealed circumferential, smooth luminal narrowing in the distal esophagus, extending up to the gastroesophageal junction over a length of 2 cm. This finding included shouldering of the proximal dilated esophagus, with contrast passing through the narrowed segment into the stomach. There was no evidence of wall irregularity or mass. Severe dilatation of the epiphrenic part of the esophagus was observed, with an out-pouching arising from the right postero-lateral part, suggestive of the epiphrenic diverticulum measuring in size 4.4 x 4.5 cm (anterior-posterior x transverse). The gastro-esophageal junction was 3 cm above the level of the left hemidiaphragm suggestive of a small hiatus hernia (Figure 1).

**Figure 2:**
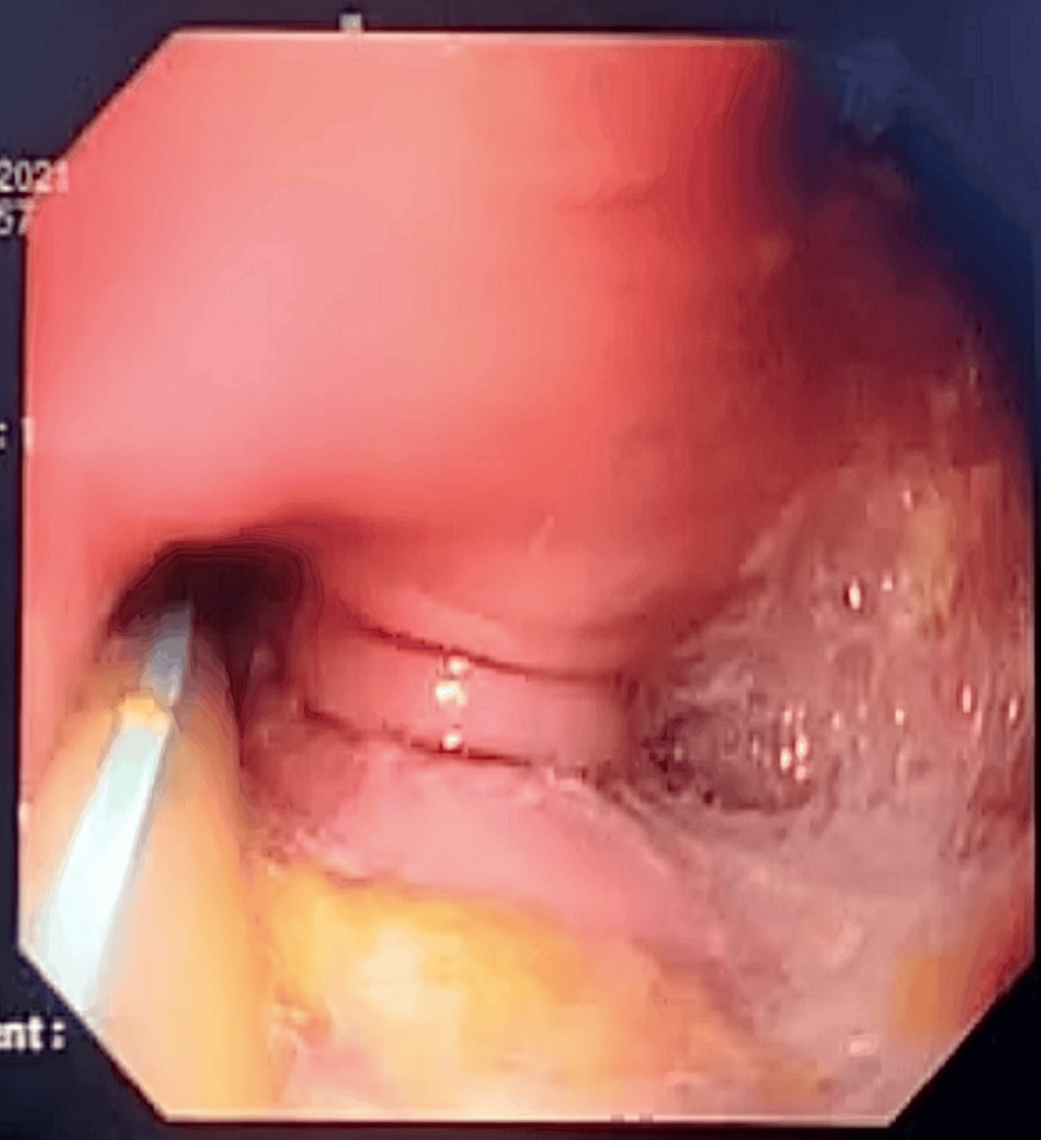
Upper gastrointestinal endoscopy image showing the esophageal diverticulum.

The upper gastrointestinal endoscopy revealed Grade D esophagitis and a dilated esophagus with a large diverticulum. The inlet of the diverticulum was located on the right wall, 35 cm from the incisors. The gastroesophageal junction was at 39 cm. Additionally, gastric antral erosions were noted and tested positive for *Helicobacter pylori* (Figure 2).

**Figure 3:**
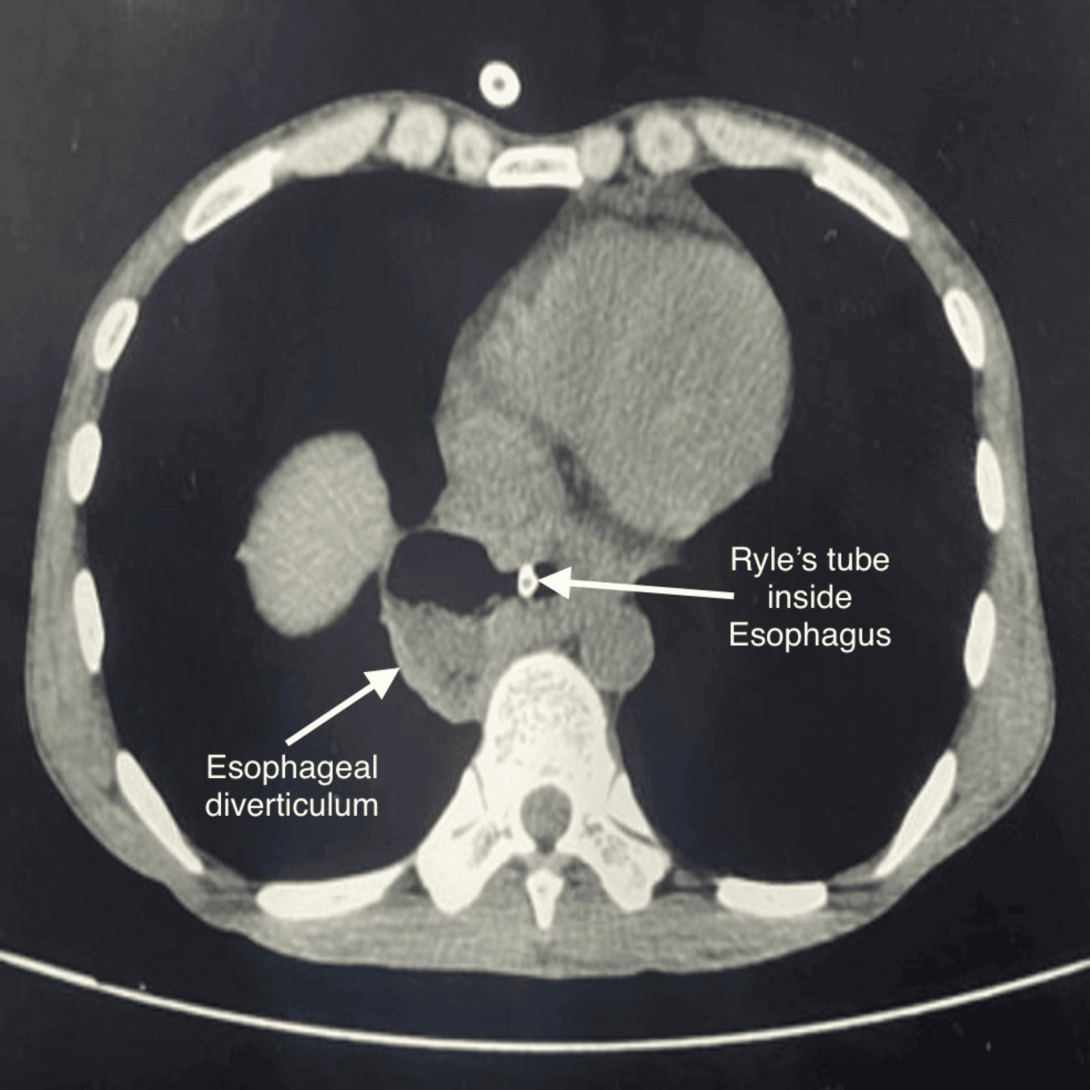
Preoperative chest computed tomography scan showing dilatation of the esophagus and an epiphrenic diverticulum measuring 4.19 x 2.93 cm.

A chest computed tomography (CT) scan showed a large epiphrenic diverticulum in the lower esophagus on the right lateral side at levels of T9 and T10 measuring 4.19 x 2.93 cm with air-fluid level noted within (Figure 3).

**Figure 4:**
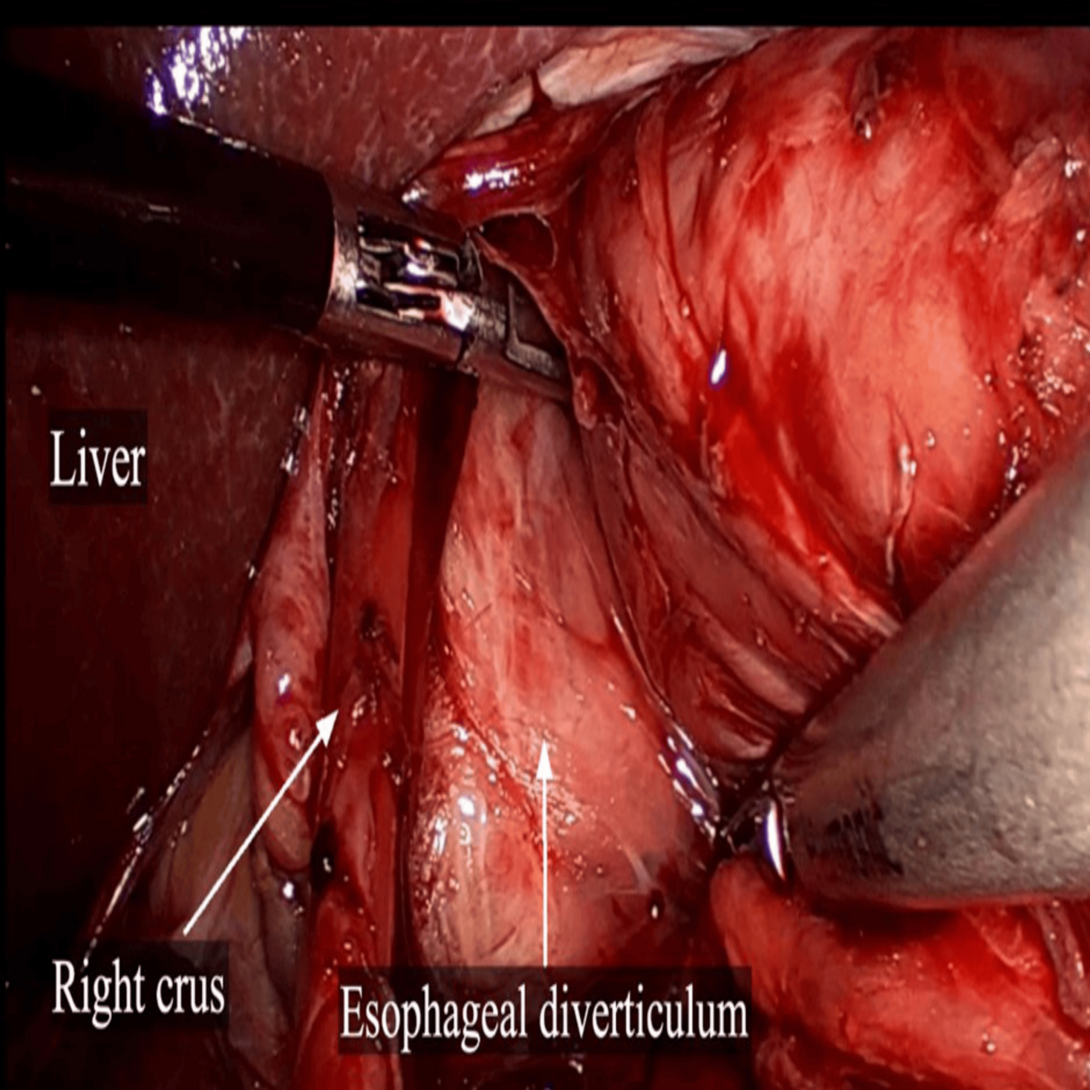
Laparoscopic view of esophageal diverticulum

**Figure 5:**
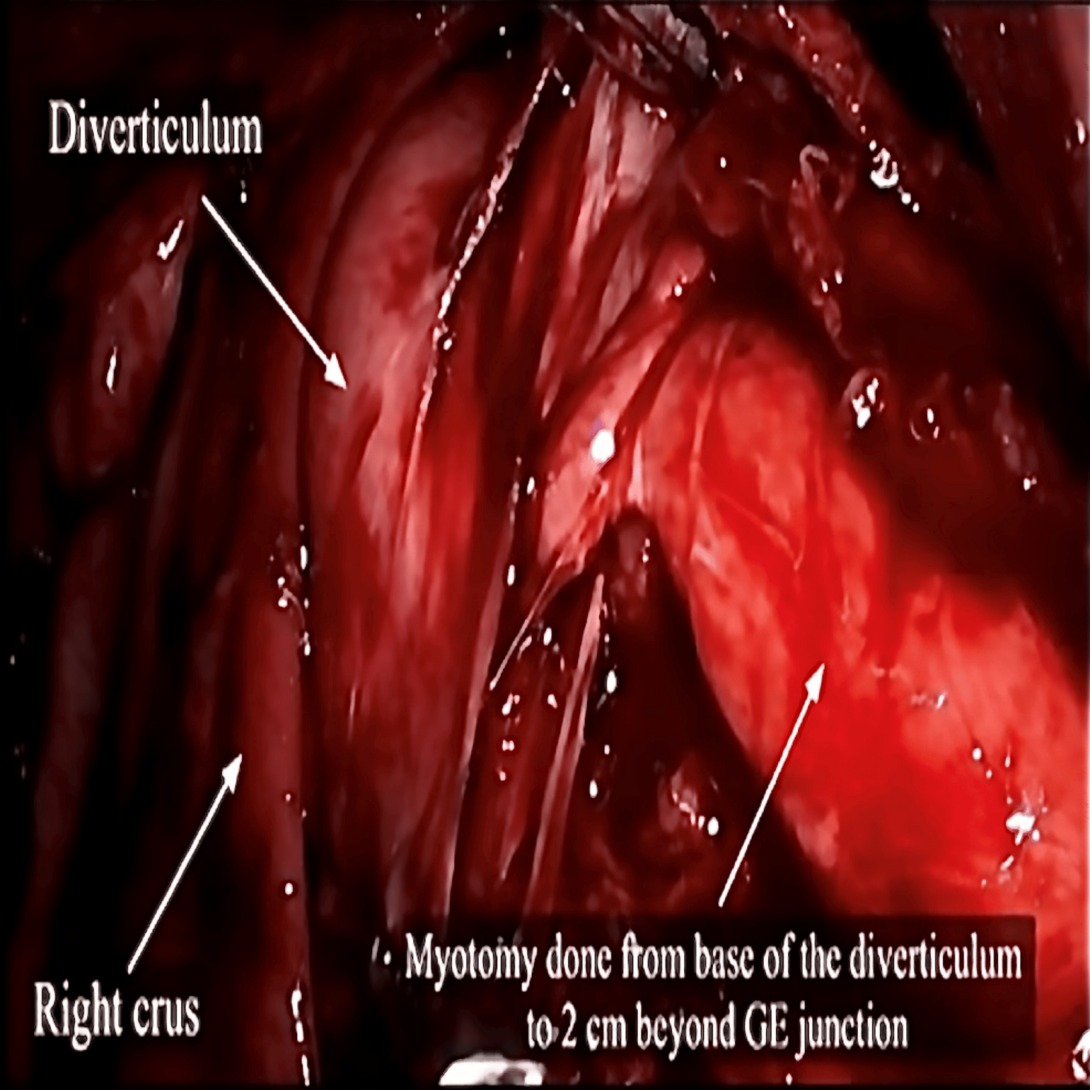
Intraoperative image showing myotomy GE: gastrointestinal endoscopy

Esophageal manometry revealed a high-pressure zone distal to the neck of the diverticulum at the lower esophageal sphincter with pressure of 70 mmHg. With the diagnosis of a large epiphrenic esophageal diverticulum, surgery was conducted after a preoperative optimization. Laparoscopic Heller’s cardiomyotomy with Dor’s fundoplication was done. Intraoperatively, the epiphrenic diverticulum was found to be broad-based just above and to the right of the gastroesophageal junction (Figure 4). Esophageal myotomy was carried out from the base of the diverticulum till 2 cm below the gastroesophageal junction (Figure 5).

**Figure 6:**
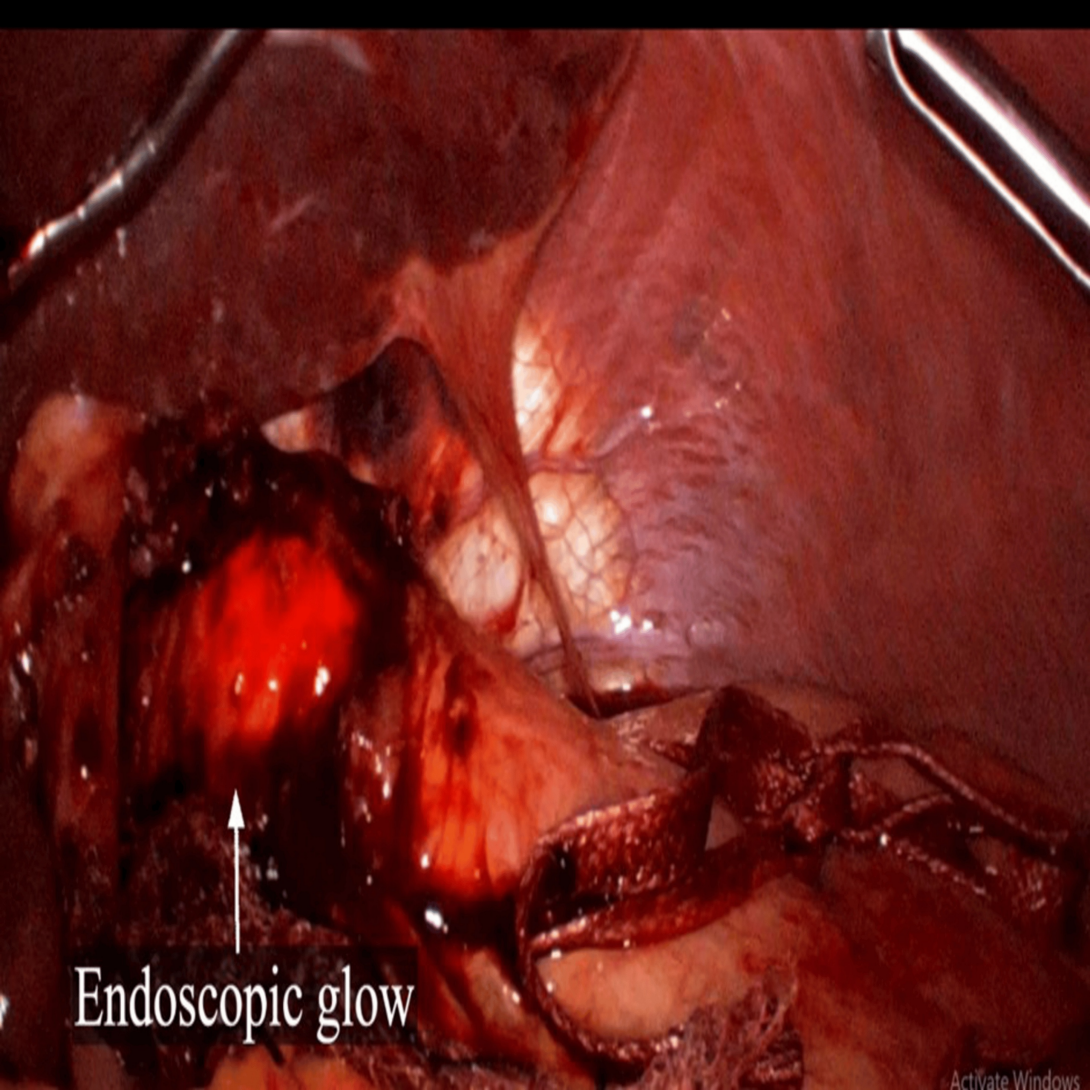
Laparoscopic view showing the glow of the endoscopic light within the esophageal lumen.

**Figure 7:**
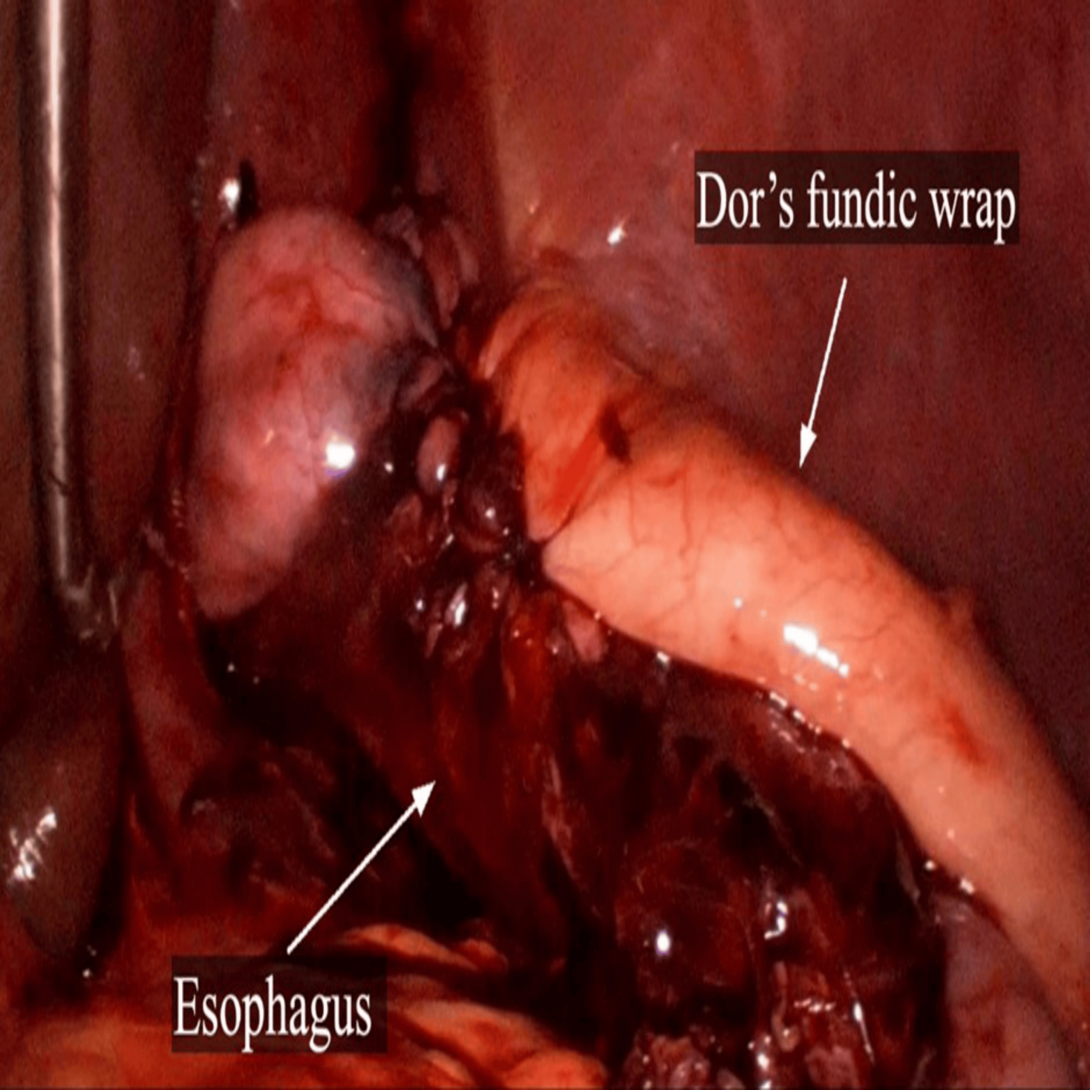
Laparoscopic view of Dor’s fundoplication wrap.

Intraoperative upper gastrointestinal endoscopy was done after the myotomy to confirm the adequacy of myotomy and mucosal integrity (Figure 6). Dor's fundoplication was done using 2-0 synthetic delayed absorbable polyglactin sutures (Figure 7). The surgical time was 190 minutes and intra-operative blood loss was 50 mL.

**Figure 8:**
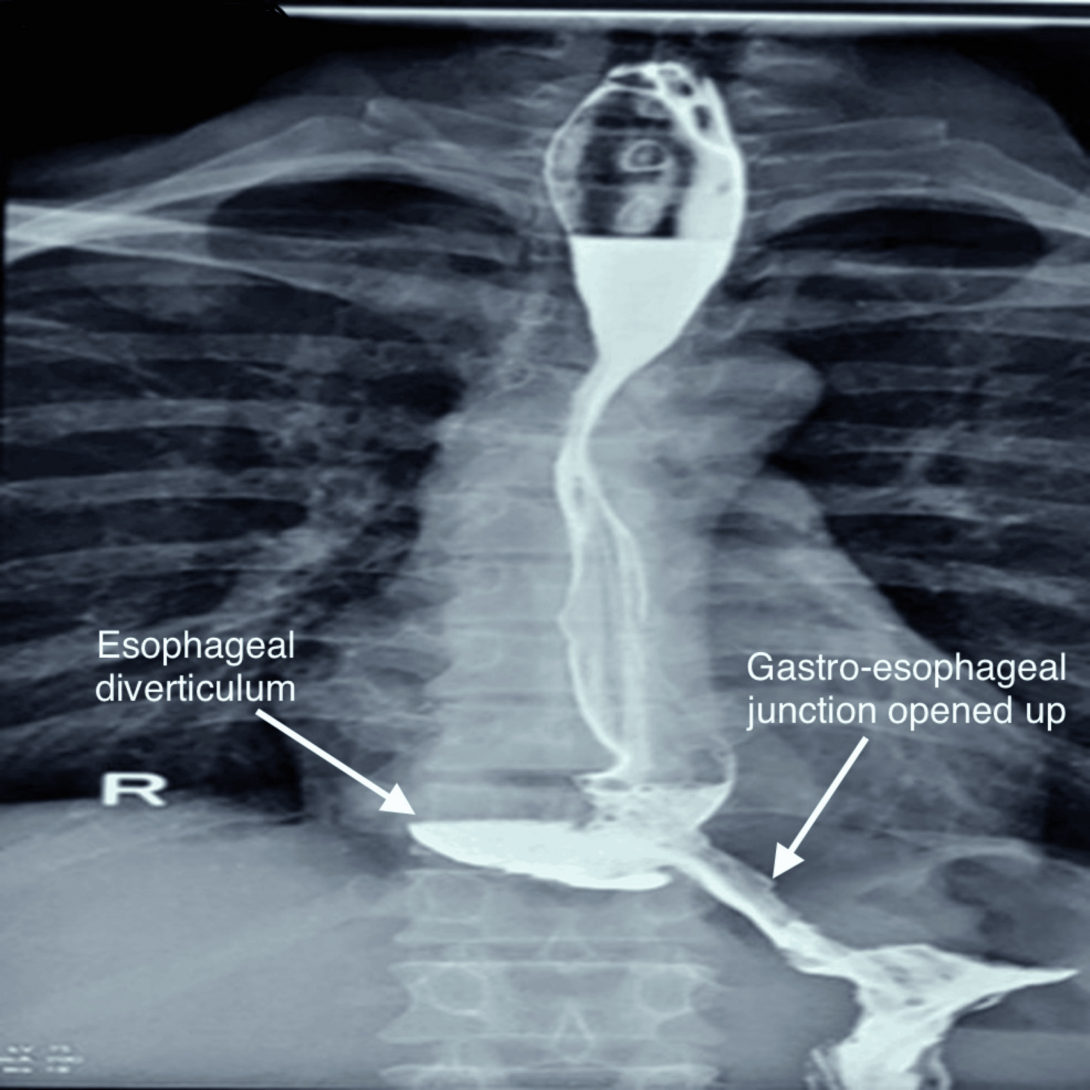
Postoperative barium swallow study showing a widely opened gastroesophageal junction with successful passage of contrast, without leakage or stenosis.

The postoperative course was uneventful; a liquid diet was started the day following surgery with rapid progression to a normal diet over the following two days. An upper gastrointestinal contrast study demonstrated good passage, with no leakage or stenosis of the esophagus (Figure 8).

At a two-year follow-up, the patient reported no dysphagia and had regained weight. Follow-up endoscopy showed that the diverticulum was persistent but the patient was free of both dysphagia and reflux.

## Discussion

Epiphrenic esophageal diverticulum is an uncommon cause of severe dysphagia. The most recent studies have shown that apart from dysphagia, the esophageal diverticulum can cause heartburn, regurgitation, chronic cough, aspiration, and pneumonia [1-3]. Yahata et al. reported that hiccups have been observed following candidial infection [4]. In untreated patients, potentially lethal complications of diverticular rupture and haematemesis have also been reported [5].

Medical and endoscopic therapies play a limited role in symptomatic patients. The mainstay of treatment in symptomatic patients is surgical. Procedures like diverticulectomy, myotomy, and fundoplications have been standard treatment methods for esophageal diverticulum. Historically, the left transthoracic approach had been a standard approach. Diverticulectomy alone is associated with an incidence of diverticular recurrence of 10-20% [6]. Diverticulectomy alone has been considered sufficient in patients with normal manometry. However, primary esophageal motility disorder is often the underlying cause and myotomy is an essential component of surgical treatment. A long myotomy has been recommended. As a long myotomy can potentially lead to reflux, an antireflux procedure is often added - usually an anterior partial wrap (Dor’s fundoplication). It also serves as a mucosal patch for inadvertent punctures during cardiomyotomy. The practice of trans-thoracic access is being replaced by a minimal access laparoscopic approach [6,7]. Peroral endoscopic myotomy (POEM) is an endoscopic approach to myotomy [8].

The diverticulum is usually managed by surgical excision. The availability of stapler technology has facilitated excision. Endoscopic staplers have made diverticulectomy possible through a minimal access approach. However, diverticulectomy has accompanying morbidity and mortality due to suture-line leaks. Recurrence after diverticulectomy is also known. Recently, a staged approach has been recommended, with laparoscopic myotomy as the initial step. Diverticulectomy is reserved for patients who do not achieve symptom relief after myotomy and continue to experience issues due to the diverticulum [9]. Intraoperative endoscopy has been reported to be useful in assessing the adequacy of myotomy [10].

In the present case, diverticulectomy was not done. Intraoperative endoscopy was carried out which ensured the sufficiency of myotomy and ruled out any mucosal breach and leak. The laparoscopic approach, avoidance of diverticulectomy, and intraoperative endoscopy enabled a quick postoperative recovery with tolerance of oral intake on the next day of surgery.

## Conclusions

Not every esophageal epiphrenic diverticulum requires diverticulectomy. Adopting a myotomy-first approach can reduce the risk of complications associated with diverticulectomy. Intra-operative endoscopy is useful to ensure a complete myotomy and rule out or identify inadvertent mucosal breaches during myotomy. Laparoscopic myotomy with anterior partial wrap without diverticulectomy enables fast tracking of postoperative recovery. Further research with an adequate sample size is needed to bolster the conclusion.
